# Vancomycin and Home Health Care

**DOI:** 10.3201/eid1110.050336

**Published:** 2005-10

**Authors:** Thomas G. Fraser, Valentina Stosor, Qiong Wang, Anne Allen, Teresa R. Zembower

**Affiliations:** * Northwestern University Feinberg School of Medicine, Chicago, Illinois, USA; † University of Illinois at Chicago School of Public Health, Chicago, Illinois, USA; ‡ Northwestern Memorial Hospital, Chicago, Illinois, USA

**Keywords:** vancomycin, home healthcare, HICPAC guidelines, outpatient parenteral antimicrobial therapy, antimicrobial resistance, research

## Abstract

The Hospital Infection Control Practices Advisory Committee published guidelines for prudent use of vancomycin to combat increasing resistance to antimicrobial drugs. Studies examining compliance with these guidelines primarily involve hospitalized patients. The growing practice of home use of antimicrobial drugs led to this retrospective cohort study that evaluated parenteral vancomycin use in patients receiving it through a homecare agency. We found that 39.2% of outpatients received vancomycin outside the guidelines, mainly because of prolonged empiric therapy, dosing convenience, and prolonged use after surgery. Patients were more likely to receive vancomycin appropriately if they were >65 years of age, had a history of malignancy, or were discharged from a medical service. In addition, obtaining wound cultures and attempting a microbiologic diagnosis led to more appropriate vancomycin use. Recommendations for prudent vancomycin use are often overlooked when selecting antimicrobial drugs for home infusion. The public health impact of this practice remains unknown.

## Abstract 2

Microbiologic diagnosis before hospital discharge and physician education may limit inappropriate vancomycin use in homecare patients.

Vancomycin is an important agent for the treatment of serious infections caused by gram-positive bacteria (*1*). Over the past 3 decades, its use has steadily increased because of increasing prevalence of β-lactam-resistant nosocomial pathogens, particularly, methicillin-resistant *Staphylococcus aureus* (MRSA) and coagulase-negative staphylococci (CoNS) (*2**,**3*). A consequence of this increased use has been the emergence and spread of vancomycin-resistant enterococci, the isolation of *S. aureus* with reduced susceptibility to glycopeptides, and, most recently, reports of infections caused by vancomycin-resistant *S. aureus* (*4**–**6*).

Antimicrobial stewardship guidelines have been developed to ensure that vancomycin is used appropriately and retains its viability in the therapeutic armamentarium. The most broadly accepted benchmark was published by the Hospital Infection Control Practices Advisory Committee (HICPAC) of the Centers for Disease Control and Prevention (*7*). These guidelines and most efforts to control use of antimicrobial drugs target the hospital setting (*8**–**12*). However, the prevalence of drug-resistant pathogens in outpatient and ambulatory settings is increasing, as demonstrated by the prevalence of penicillin-resistant pneumococci and recent emergence of community-onset MRSA (*13**,**14*). With an increasing number of patients receiving home infusions of antimicrobial drugs, the appropriateness of choices of drugs for outpatients warrants scrutiny. Guidelines for the administration of outpatient parenteral antibiotic therapy (OPAT) noted this and encouraged adherence to HICPAC guidelines (*15*).

We conducted a retrospective cohort study of patients discharged from an academic medical center to complete a course of intravenous vancomycin at home. The main objectives were to describe the epidemiology of outpatients receiving vancomycin through a home healthcare agency, determine the appropriateness of outpatient vancomycin prescriptions according to HICPAC guidelines, and examine factors associated with outpatient vancomycin use that conformed to HICPAC guidelines.

## Methods

### Study Setting and Patient Population

Northwestern Memorial Hospital (NMH) is a 725-bed teaching hospital in Chicago, Illinois. Northwestern Memorial Home Health Care, Inc. (NMHHC), the home healthcare agency affiliated with NMH, receives >200 annual referrals for home infusion of antimicrobial agents.

This study included all inpatients at NMH referred to NMHHC to complete a course of intravenous vancomycin therapy from December 1997 to April 2002. Patients were excluded if they were <16 years of age, admitted to the hospital already receiving vancomycin, discharged to any other facility, or received care from another homecare agency before referral to NMHHC. For patients with multiple referrals to NMHHC for vancomycin therapy, only their first treatment episode was included. During this study, although vancomycin use guidelines were published and distributed within NMH, no formal enforcement policy existed within the hospital or homecare setting. The institutional review board of Northwestern University reviewed and approved the study protocol.

### Clinical Data

All data were originally collected as part of routine patient care. For this study, clinical data were abstracted retrospectively by review of existing inpatient medical records, home health referral forms, and the inpatient pharmacy database. The data abstractor had no part in the original data collection. The following data were abstracted: demographic information, length of hospital stay, admitting service, insurance status, allergy to β-lactam antimicrobial drugs, level of serum creatinine on the day of discharge, history of end-stage renal disease requiring dialysis, infectious diseases consultation, use of vancomycin in the hospital, reason(s) for vancomycin use, and discharge diagnoses per ICD-9 codes. ICD-9 codes were used to calculate a mean Charlson comorbidity score for each patient (*16**,**17*). With 1 exception, the presence of infectious syndromes was determined by review of ICD-9 diagnoses. A diagnosis of bloodstream infection was assigned if multiple positive blood cultures were documented, regardless of coded diagnoses. Because of the retrospective nature of the evaluation, all recorded allergies to β-lactam antimicrobial drugs were considered potentially serious.

### Microbiologic Data

The microbiology records spanning the length of the hospitalization for each patient were reviewed. A microbiologic evaluation occurred if cultures were obtained that reasonably corresponded to the infectious diagnosis requiring the use of vancomycin. Record review focused on collection of cultures from blood, other sterile sites, urine, sputum, intravenous catheters or other foreign bodies, and wounds or tissues. Bacterial isolates that were specifically recorded were gram-positive organisms whose treatment might prompt or warrant the use of vancomycin, including methicillin-susceptible *S. aureus*, MRSA, CoNS, streptococci, ampicillin-resistant or -susceptible enterococci, and *Corynebacterium jeikeium*.

### Evaluation of Vancomycin Use

HICPAC guidelines served as the basis for determining whether patients received parenteral vancomycin per guidelines or outside guidelines (Table 1). The guidelines pertaining to prophylaxis for endocarditis (1C), surgical procedures (1D and 2A), and low-birthweight infants (2G) did not apply and were disregarded.

**Table 1:** HICPAC guidelines for prudent use of parenteral vancomycin*

1) Situations in which use of vancomycin is appropriate
A) Treatment of serious infections caused by β-lactam-resistant, gram-positive organisms
B) Treatment of infections caused by gram-positive microorganisms in patients with serious allergies to β-lactam antimicrobial agents
C) Prophylaxis, as recommended by the American Heart Association, after certain procedures in patients at high risk for endocarditis
D) Prophylaxis for major surgical procedures involving implantation of prosthetic materials or devices at institutions that have a high rate of infections caused by MRSA or methicillin-resistant *Staphylococcus epidermidis*
2) Situations in which use of vancomycin should be discouraged
A) Routine surgical prophylaxis, unless patient has life-threatening allergy to β-lactam antimicrobial drugs
B) Empiric antimicrobial therapy for febrile neutropenic patient, unless evidence indicates patient has infection caused by gram-positive microorganisms and prevalence of MRSA infections in hospital is substantial
C) Treatment in response to single blood culture positive for coagulase-negative staphylococci, if other blood cultures taken during same timeframe are negative
D) Continued empiric use for presumed infections in patients whose cultures are negative for β-lactam-resistant gram-positive microorganisms
E) Systemic or local (e.g., antimicrobial drug lock therapy)† prophylaxis for infection or colonization of intravascular catheters
F) Eradication of MRSA colonization
G) Routine prophylaxis for very-low-birthweight infants
H) Routine prophylaxis for dialysis patients
I) Treatment (chosen for dosing convenience) of infections caused by β-lactam-sensitive, gram-positive microorganisms in patients with renal failure

*Summarized from reference 7. HICPAC, Hospital Infection Control Practices Advisory Committee; MRSA, methicillin-resistant *Staphylococcus aureus*. †Instilling a high concentration of antimicrobial drug to which organism is susceptible into lumen of catheter in attempt to sterilize it.

In addition, vancomycin use was determined to fall outside HICPAC guidelines for the following situations: 1) treatment of CoNS from superficial wound swabs, or respiratory or urine specimens unless they occurred in the setting of bacteremia; 2) dosing convenience defined as initial treatment with a β-lactam antimicrobial drug during hospitalization with a therapeutic change to vancomycin within 24 h of discharge that was not dictated by culture results or allergy; 3) prolonged administration of an antimicrobial agent after implantation of prosthetic materials; 4) treatment of cellulitis without identification of a β-lactam-resistant pathogen (additionally, the empiric switch to vancomycin because of slow resolution of cellulitis was considered noncompliant use); and 5) ongoing treatment of infection in a patient with a history of MRSA colonization in the absence of a diagnostic culture. If the use of vancomycin met >1 of these specified criteria, each was included in data collection.

### Statistical Analysis

Data were collected on a standardized form and entered onto spreadsheets (Excel 2000, Microsoft Corporation, Redmond, WA, USA). To evaluate predictors for compliance with vancomycin use guidelines, discrete variables were described by percentages and compared by using chi-square or Fisher exact tests as appropriate. Continuous variables were described by means and evaluated by using Student t test. Variables with a p value <0.05 by univariate analysis were evaluated by stepwise logistic regression for inclusion in the final model. SAS version 8.2 for personal computers (SAS Institute Inc., Cary, NC, USA) was used for statistical analysis.

## Results

During the study period, NMHHC received 323 patient referrals for continuation of parenteral vancomycin therapy after hospitalization. The records of 27 patients (8.4%) could not be located and were not included in the study. Thus, the final analysis included 296 patients. Table 2 summarizes the criteria that determined whether vancomycin was prescribed within HICPAC guidelines. One hundred eighty patients (60.8%), 5 of whom met >1 criteria for appropriate use, received vancomycin within guidelines. A total of 118 (65.6%) were treated for infections caused by β-lactam-resistant, gram-positive bacteria. Sixty-seven patients (37.2%) received vancomycin for a reported allergic reaction to β-lactam antimicrobial drugs. Although only the first referral for home vancomycin was analyzed for each patient, 44 (14.9%) were referred multiple times to receive vancomycin as outpatients (2–8 referrals per patient) during this study.

**Table 2:** Comparison of HICPAC guidelines with home infusion use of vancomycin in 296 patients*

	No. (%)
Manner in which vancomycin use met guidelines	180 (60.8)
Treatment of infections with β-lactam-resistant, gram-positive bacteria	118 (65.6)
Treatment of gram-positive infections in patients with allergies to β-lactam agents	67 (37.2)
Manner in which vancomycin use did not meet guidelines	116 (39.2)
Continued empiric vancomycin use in patients with negative or no cultures	84 (72.4)
Use of vancomycin for dosing convenience	18 (15.5)
Prolonged administration of antimicrobial drugs after implantation of prosthetic materials or devices	13 (11.2)
Treatment of a single blood culture showing coagulase-negative staphylococci	9 (7.8)

*Some patients fulfilled >1 criteria. HICPAC, Hospital Infection Control Practices Advisory Committee.

Of the 296 patients, 116 (39.2%), 8 of whom met >1 criteria, received vancomycin outside HICPAC guidelines. Eighty-four (72.4%) cases were for continued empiric treatment of presumed infections in patients whose cultures were negative or not obtained. This practice was prevalent across all services. Dosing convenience led to the use of the drug in 18 (15.5%) patients, 12 (67%) of whom were admitted to a medical service. In 13 patients (11.2%), home-infusion vancomycin was continued after major surgical procedures involving implanted devices. This practice occurred exclusively in orthopedic and neurosurgery services. Finally, in 9 patients (7.8%), vancomycin was used to treat infection with a CoNS isolate from a single blood culture.

Demographic and clinical characteristics are shown in Table 3. Patients whose use of vancomycin followed guidelines were older than those whose use did not follow guidelines (mean age 53.6 years vs. 48.9 years, p = 0.016). No significant differences were noted in sex or ethnicity, although African-Americans showed a trend toward receiving vancomycin within guidelines (p = 0.054). Appropriate vancomycin use was more likely after a longer mean hospital stay (12.2 days vs. 9.5 days, p = 0.007). No significant differences were noted in the mean Charlson comorbidity score or frequency of diagnosed coexisting medical conditions between the 2 groups with the exception of a history of malignancy (21.7% vs. 10.3%, p = 0.012) among patients who received vancomycin according to guidelines. Insurance status did not differ between groups.

**Table 3:** Demographic and clinical characteristics of 296 patients referred for home infusions of vancomycin from December 1997 through May 2002*

Characteristic	Use per guidelines, N = 180	Use outside guidelines, N = 116	p value†
Mean age, years (range)	53.6 (19–90)	48.9 (19–86)	0.016
Male, no. (%)	109 (60.6)	76 (65.5)	0.389
Ethnicity, no. (%)			
Caucasian	100 (55.6)	75 (64.7)	0.120
African-American	52 (28.9)	22 (19.0)	0.054
Asian	3 (1.7)	0	0.283
Hispanic	7 (3.9)	2 (1.7)	0.490
Other	18 (10.0)	15 (12.9)	0.434
Mean length of stay, days (range)	12.2 (2–52)	9.5 (2–67)	0.007
Coexisting conditions, no. (%)			
Diabetes mellitus	47 (26.1)	28 (24.1)	0.703
Malignancy	39 (21.7)	12 (10.3)	0.012
Spinal cord injury	28 (15.6)	13 (11.2)	0.290
Decubitus ulcer	31 (17.2)	11 (9.5)	0.063
Acute renal failure	15 (8.3)	6 (5.2)	0.301
ESRD	5 (2.8)	1 (0.9)	0.409
Immunocompromised status	10 (5.6)	8 (6.9)	0.637
HIV	3 (1.7)	1 (0.9)	1.000
Charlson score, mean (range)	1.4 (0–8)	1.1 (0–14)	0.217
Insurance status, no. (%)	176 (97.8)	116 (100)	0.158
Private	121 (67.2)	86 (74.1)	0.205
Medicare	74 (41.1)	36 (31.0)	0.080
Medicaid	48 (26.7)	25 (21.6)	0.319
Discharging service, no. (%)			
Medical	108 (60.0)	44 (37.9)	<0.001
General medicine	66 (36.7)	29 (25.0)	0.036
Medicine subspecialties	29 (16.1)	10 (8.6)	0.063
Hematology/oncology	13 (7.2)	5 (4.3)	0.306
Surgical	67 (37.2)	69 (59.5)	<0.001
General surgery	2 (1.1)	4 (3.5)	0.215
Transplant surgery	5 (2.8)	6 (5.2)	0.350
Vascular surgery	14 (7.8)	7 (6.0)	0.569
Orthopedics/neurosurgery	31 (17.2)	41 (35.3)	<0.001
Other surgical subspecialties	15 (8.3)	11 (9.5)	0.733
Other hospital services	5 (2.8)	3 (2.6)	1.000
Consultation by infectious diseases, no. (%)	85 (47.2)	58 (50.0)	0.641

*ESRD, end-stage renal disease requiring dialysis; immunocompromised status, immunocompromised from causes other than HIV. †Values <0.05 were considered significant.

Compliance with HICPAC guidelines varied according to the inpatient prescribing service. Appropriate prescriptions for vancomycin were more likely to be preceded by discharge from a medical service (60.0% vs. 37.9%, p<0.001). This finding was true both for discharges from general medicine and medical subspecialty services, with the exception of hematology/oncology. More episodes of vancomycin infusion outside guidelines followed discharge from a surgical service (59.5% vs. 37.2%, p<0.001), namely, orthopedic and neurosurgery services (35.3% vs. 17.2%, p<0.001). Inpatient consultation by an infectious diseases specialist did not affect the appropriateness of home vancomycin prescriptions by managing services (p = 0.641).

The infection diagnoses of patients referred for home infusions of vancomycin are outlined in Table 4. Patients were more likely to receive vancomycin per guidelines in the setting of bloodstream (33.9% vs. 13.8%, p<0.001) and urinary tract infections (20% vs. 11.2%, p = 0.042). The microbiologic investigations undertaken and the organisms identified during hospitalization are delineated in Table 5. Appropriate use of vancomycin was more likely to follow an attempt to make a microbiologic diagnosis (96.1% vs. 77.6%, p<0.001). More blood, urine, and wound cultures were obtained in this group, and the number of cultures obtained was higher when vancomycin was used appropriately.

**Table 4:** Infection diagnoses in patients referred for home infusions of vancomycin

Diagnosis*	Use per guidelines, no. (%), N = 180	Use outside guidelines, no. (%), N = 116	p value†
Skin or soft tissue infection	89 (49.4)	51 (44.0)	0.356
Osteomyelitis or septic arthritis	30 (16.7)	25 (21.6)	0.294
Postoperative wound infection	38 (21.1)	19 (16.4)	0.310
Orthopedic device-related infection	5 (2.8)	7 (6.0)	0.227
Central nervous system infection‡	3 (1.7)	6 (5.2)	0.161
Urinary tract infection	36 (20.0)	13 (11.2)	0.042
Pneumonia	9 (5.0)	1 (0.9)	0.095
Bloodstream infection	61 (33.9)	16 (13.8)	<0.001
Vascular device infection	21 (11.7)	10 (8.6)	0.398
Infective endocarditis	8 (4.4)	5 (4.3)	0.956

*Some patients had >1 diagnosis. †Values <0.05 were considered significant. ‡Includes shunt infections.

**Table 5:** Microbiologic investigations and results for home infusions of vancomycin*

Investigation or result	Use per guidelines, no. (%), N = 180	Use outside guidelines, no. (%), N = 116	p value†
Microbiologic diagnostic attempt	173 (96.1)	90 (77.6)	<0.001
Cultures by site			
Blood	137 (76.1)	74 (63.8)	0.022
Sterile site	25 (13.9)	11 (9.5)	0.258
Urine	96 (53.3)	48 (41.4)	0.045
Sputum	17 (9.4)	6 (5.2)	0.180
Wound	96 (53.3)	45 (38.8)	0.015
Other culture	15 (8.3)	11 (9.5)	0.733
>1 culture	136 (75.6)	65 (56.0)	<0.001
Bacterial isolates			
MRSA	81 (45.0)	2 (1.7)‡	§
Coagulase-negative staphylococci	59 (32.8)	20 (17.2)	
Ampicillin-resistant enterococci	3 (1.7)	0	
Methicillin-susceptible *S. aureus*	16 (8.9)	19 (16.4)	
Other streptococci and enterococci	52 (28.9)	18 (15.5)	
*Corynebacterium jeikeium*	2 (1.1)	0	
Culture considered contaminated	20 (11.1)	20 (17.2)	

*MRSA, methicillin-resistant *Staphylococcus aureus*. †Values <0.05 were considered significant. ‡The 2 patients outside the guidelines with an MRSA culture included 1 patient with MRSA in the urine but no diagnosis of a urinary tract infection, and 1 with a positive intravascular catheter tip culture but no evidence of infection. §A p value is not included because infection with these isolates was 1 factor used to determine whether vancomycin was given per guidelines.

Results of the multivariate analysis are shown in Table 6. Patients <65 years of age were less likely to receive appropriate vancomycin (odds ratio [OR] 0.50, 95% confidence interval [CI] 0.26–0.94). Appropriate use of vancomycin was more likely to occur after discharge from a medical service rather than a surgical service (OR 2.62, 95% CI 1.53–4.48). Although discharge from a hematology/oncology service was not associated with appropriate use of vancomycin, patients with a history of malignancy were more likely to receive vancomycin within HICPAC guidelines (OR 3.02, 95% CI 1.40–6.53). Obtaining a wound culture was associated with appropriate use of vancomycin (OR 2.08, 95% CI 1.19–3.64). Finally, patients who underwent any microbiologic evaluation were more likely to receive appropriate vancomycin through home care (OR 5.93, 95% CI 2.26–15.54).

**Table 6:** Factors associated with appropriate use of vancomycin by multivariate analysis using stepwise logistic regression analysis

Variable	Odds ratio (95% CI)*	p value
Attempt at a microbiologic diagnosis	5.93 (2.26–15.54)	0.0003
Discharge from a medical service	2.62 (1.53–4.48)	0.0004
History of a malignancy	3.02 (1.40–6.53)	0.0050
Obtaining a wound culture	2.08 (1.19–3.64)	0.0107
Age <65	0.50 (0.26–.094)	0.0321

*CI, confidence interval.

## Discussion

This study examined a large group of patients referred for home infusions of vancomycin over a 5-year period and applied established guidelines to determine if outpatient use conformed to a widely accepted benchmark. A total of 39.2% of the prescriptions were given outside guidelines. Several authors have applied these HICPAC guidelines to evaluate inpatient use of vancomycin and found the incidence of outside guidelines use to range from 36% to 79% (*11**,**12**,**18**,**19*). Our study, however, is the first to critically evaluate the appropriateness of vancomycin in the outpatient setting.

The most common reason for outside guidelines use of vancomycin was continuation of empiric therapy in patients without a culture-defining indication. Singer et al. have found similar results in hospitalized patients (*12*). In contrast, other studies found that the most common reasons for inappropriate inpatient prescriptions for vancomycin were surgical prophylaxis and failure to modify prescriptions for antimicrobial drugs based on culture results (*19**,**20*).

We found that the other reasons for vancomycin use outside guidelines were dosing convenience, prolonged use after surgical procedures, and treatment of CoNS isolated from a single blood culture. Use for dosing convenience is likely underestimated (15.5%) because of the conservative definition used in this retrospective analysis. The incidence of vancomycin use for prolonged periods after implantation of devices and for the treatment of CoNS from a single blood culture was less than the incidence among inpatients (*12**,**19*). This incidence may reflect that continuing vancomycin for these indications is more convenient in the inpatient setting and that physicians are likely to reevaluate the true need for outpatient vancomycin in these circumstances.

Examined data showing the prescribing patterns of physicians demonstrate that patients discharged from a medical service are more likely to receive vancomycin appropriately. Of surgical subspecialists, orthopedic and neurosurgeons were more likely to prescribe vancomycin outside guidelines. These prescribing differences are consistent with the findings of inpatient vancomycin use evaluations (*21**–**24*). Although patients with a history of malignancy received vancomycin according to HICPAC guidelines, hematology/oncology was the only medical service not associated with appropriate use. These results suggest that the vancomycin-prescribing practices of certain subspecialists offer the opportunity for education regarding the existence of and rationale for such guidelines and targeted intervention to reduce unnecessary outpatient vancomycin usage (*25*). Only 6 patients with end-stage renal disease received vancomycin through homecare. Intuitively, one might expect more vancomycin use in this patient population; however, this finding probably reflects that these patients receive vancomycin during hemodialysis and, thus, do not require referral to home health. In contrast to other studies, consultation by infectious diseases physicians did not impact compliance (*26**–**28*). This finding warrants further examination to determine if infectious diseases physicians recommend vancomycin for use outside of HICPAC guidelines or if their recommendations are disregarded.

If a microbiologic evaluation was attempted, vancomycin use was more likely to follow guidelines. Obtaining wound cultures was also associated with appropriate use. A thorough microbiologic evaluation aids in clinical decision making. When clinicians have culture and susceptibility results, they are more likely to use vancomycin appropriately, particularly for patients with skin and soft tissue infections.

Patients >65 years of age were more likely to receive vancomycin per guidelines. The reasons for this are unclear but were not impacted by insurance status. This finding probably reflects that patients referred for intravenous antimicrobial drugs through homecare either have insurance that will reimburse for the service or have the ability to pay for the drugs.

This study had several limitations because of its retrospective nature. A substantial number of patients were classified in the compliant group on the basis of a reported allergy to β-lactam drugs. Because we were unable to determine the nature of reported allergies to penicillin, all allergies were assumed to be serious in nature. Thus, this study overestimates appropriate vancomycin use for this purpose. Another limitation of this analysis is the inability to account for the impact of vancomycin courses patients may have received before this study. Finally, this study does not address the financial consideration that influenced the choice of antimicrobial drug. Other investigators have explored this issue and found that the costs of outpatient vancomycin therapy are substantial (*29*). The patients in this study were preselected to the extent that they were able to receive vancomycin at home.

HICPAC guidelines were developed to promote judicious use of vancomycin in an attempt to curtail the spread of vancomycin-resistant enterococci and forestall the development of *S. aureus* with reduced susceptibility to glycopeptides. Although these guidelines were initially applied to the inpatient setting, the OPAT guidelines have recommended that they also apply to outpatients receiving vancomycin. Apart from vancomycin, however, the OPAT guidelines lack information regarding choices of antimicrobial drugs for outpatients. In addition, they do not clearly prioritize conscientious use of antimicrobial drugs above other considerations, such as cost and dosing convenience, when choosing outpatient therapy. These issues need to be addressed as the emergence and spread of antimicrobial-resistant gram-positive pathogens in the community continue to increase.

One in 1,000 patients in the United States is estimated to receive outpatient infusion of antimicrobial drugs each year (*15*). The trend toward increased inpatient acuity and shorter hospital stay will undoubtedly increase this practice. Our study on first-time referrals from 1 tertiary care hospital to its homecare agency represents only a subset of vancomycin use in the community. The propensity for readmissions and repeated referrals of these chronically ill patients must be considered when analyzing the impact of outpatient vancomycin use. In addition, vancomycin administered by other homecare agencies, extended care facilities, outpatient infusion centers, and outpatient dialysis centers all contribute to its burgeoning use outside the hospital. Our study indicates that further investigations into the consequences of this practice on individual persons and the community are warranted. Do the favorable pharmacokinetics and economic attributes of vancomycin that make it attractive for home infusion outweigh the potential consequences of unnecessarily broad-spectrum gram-positive coverage? Further studies are needed to address these issues if we are to understand the dynamics of resistant pathogens in the community and the overall emergence and spread of antimicrobial resistance.
